# Plant Immunometabolism: Metabolic Reprogramming Linking Developmental Signaling and Defense Metabolites

**DOI:** 10.3390/ijms27083635

**Published:** 2026-04-19

**Authors:** Wajid Zaman, Asma Ayaz, Adnan Amin

**Affiliations:** 1 Department of Life Sciences, Yeungnam University, Gyeongsan 38541, Republic of Korea; wajidzaman@yu.ac.kr; 2 Faculty of Sports Science, Ningbo University, Ningbo 315211, China

**Keywords:** plant immunometabolism, metabolic reprogramming, plant defense signaling, secondary metabolites, plant metabolomics, stress adaptation, crop resilience

## Abstract

Plant metabolism is essential for coordinating growth, development, and defense under changing environmental conditions. Plants continuously adjust their metabolic pathways to balance resource allocation between growth and immune responses. Under stress, metabolic reprogramming redirects energy and resources toward the production of defense compounds and activation of immune signaling pathways. These changes involve complex interactions among primary metabolism, specialised metabolites, and regulatory networks, including calcium signaling, reactive oxygen species, and phytohormones. Advances in metabolomics and multi-omics technologies have improved understanding of the metabolic control of plant immunity; however, knowledge remains fragmented, and an integrated framework linking metabolism, development, and defense is still emerging. This review examines plant immunometabolism by highlighting the dynamic relationships between metabolic networks and immune functions during development and stress. It discusses pathways that influence growth, stress-induced metabolic shifts linked to defense, and how signaling interacts with metabolism. Progress in metabolomics, transcriptomics, proteomics, and computational modeling that supports systems-level analysis of plant metabolism is also summarized. In addition, potential applications in agriculture and biotechnology, including metabolic engineering, genome editing, and metabolomics-based breeding, are considered in relation to crop resilience. By integrating metabolism, signaling, and systems biology, this review provides a broad perspective on how metabolic reprogramming shapes the growth–defense trade-off in plants and outlines future directions for developing climate-resilient crops.

## 1. Introduction

Plant survival depends on the ability to continuously adjust metabolic processes in response to developmental cues and environmental challenges [1]. Plant metabolism provides the biochemical foundation for cellular growth, energy production, and biosynthesis of structural and signaling molecules [2]. These metabolic networks are tightly connected with developmental programs, allowing plants to coordinate resource allocation during processes such as organ formation, flowering, and tissue differentiation [3]. At the same time, plants must maintain the capacity to rapidly activate defense responses against pathogens and environmental stressors [4].

When plants encounter stress conditions, they undergo large-scale metabolic reprogramming. Energy and metabolic fluxes are redirected toward defense-related pathways that produce antimicrobial compounds, signaling molecules, and structural barriers [5]. This metabolic shift enables plants to mount effective immune responses while maintaining basic cellular functions [6]. Key defense metabolites include phenylpropanoids, flavonoids, terpenoids, and alkaloids, which contribute to pathogen resistance and environmental stress tolerance [7].

Plant immune responses are regulated by complex signaling networks involving calcium signaling, reactive oxygen species (ROS), and phytohormones such as salicylic acid, jasmonic acid, and ethylene [8]. These signaling pathways interact with metabolic networks to coordinate the biosynthesis of defense metabolites and to regulate systemic immune responses across plant tissues [9]. Increasing evidence suggests that plant metabolism not only supports immune responses but also contributes more broadly to determining how plants respond to environmental challenges, including both biotic and abiotic stresses.

Recent advances in metabolomics and multi-omics technologies have greatly expanded our ability to study plant metabolic responses at the systems level. High-throughput analytical platforms such as liquid chromatography–mass spectrometry (LC-MS), gas chromatography–mass spectrometry (GC-MS), and nuclear magnetic resonance (NMR) spectroscopy allow comprehensive profiling of plant metabolites under diverse environmental conditions [10]. Integration of metabolomics with transcriptomics and proteomics provides new insights into the regulatory networks controlling plant metabolic reprogramming [11]. Despite significant progress in plant metabolic research, current knowledge remains fragmented across multiple disciplines. Studies often focus on either metabolic pathways or signaling networks, while the integration between plant development and immune responses remains less explored [12]. The concept of plant immunometabolism has increasingly been recognized in the literature as a useful framework for understanding how metabolic networks interact with immune signaling pathways to regulate plant growth, defense, and stress responses [13,14].

Plant survival requires balancing metabolic resources between growth and defense [15]. Activation of immune responses demands substantial metabolic investment because plants must synthesise antimicrobial compounds, signaling molecules, and structural barriers. Consequently, defense activation often redirects carbon, nitrogen, and energy away from developmental processes [16]. This physiological constraint is widely described as the growth–defense trade-off, a central concept in plant biology [17]. Understanding how metabolic networks regulate this balance is essential for improving crop productivity under environmental stress conditions.

This review synthesises current knowledge on metabolic reprogramming that links plant development and defense responses. We discuss key metabolic pathways governing plant growth, explore stress-induced metabolic shifts during immune activation, and examine the integration of signaling networks with metabolic regulation. Furthermore, we highlight recent advances in metabolomics and systems biology approaches that enable comprehensive analysis of plant metabolic responses. Finally, we discuss potential applications of plant immunometabolism in crop improvement and outline future research directions for developing resilient agricultural systems.

## 2. Metabolic Networks Governing Plant Development

**Figure 1:**
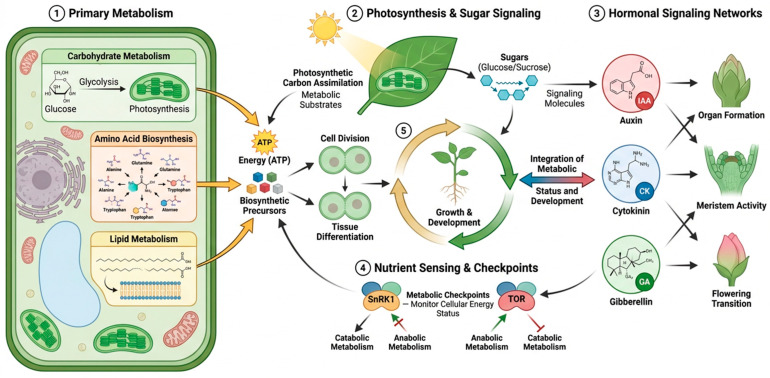
Metabolic regulation of plant development through primary metabolic pathways and hormonal signaling networks. Conceptual schematic overview of how primary metabolism supports plant growth and development. Carbon assimilation, sugar metabolism, amino acid biosynthesis-related nitrogen metabolism, and lipid metabolism provide energy and building blocks for cell proliferation, organ formation, and developmental transitions, while hormone signaling and nutrient-sensing pathways coordinate metabolic status with growth outputs. The depicted pathways and hormone structures are illustrative and representative, and do not represent a complete regulatory network. Created in BioRender. Zaman, W. (2026) https://BioRender.com/04tpr26.

Plant development relies on highly coordinated metabolic networks that regulate energy production, biosynthesis, and signaling processes. Primary metabolic pathways generate the carbon skeletons, reducing power, and ATP required for cellular proliferation and tissue differentiation [18]. These pathways integrate with developmental signaling systems to control organ formation, meristem activity, and developmental transitions. Carbon metabolism, nitrogen assimilation, and lipid biosynthesis collectively determine the availability of metabolic resources required for plant growth [19]. In addition, metabolic status influences developmental programs through nutrient-sensing pathways and hormone-mediated signaling networks [20]. Recent studies demonstrate that plant developmental plasticity is strongly influenced by metabolic flux, which determines how plants allocate resources between growth, maintenance, and stress preparedness [21,22]. These regulatory interactions form the metabolic foundation that links plant physiology with developmental regulation (Figure 1).

### 2.1. Primary Metabolism in Plant Growth

Primary metabolism provides the essential biochemical framework supporting plant growth and cellular development. Photosynthesis drives carbon assimilation through the Calvin–Benson cycle, producing triose phosphates that serve as precursors for sucrose and starch biosynthesis [23]. Sucrose is the major transportable carbon source in plants and functions as both an energy carrier and a signaling molecule that regulates gene expression [24]. Studies in *Arabidopsis thaliana* have demonstrated that sucrose signaling regulates transcription factors controlling meristem activity and leaf morphogenesis [25]. Mutations affecting sucrose transporters or carbohydrate metabolism often lead to severe developmental defects, highlighting the importance of sugar distribution in plant growth [26]. In addition, glucose sensing mediated by hexokinase functions as a metabolic signaling mechanism that links carbon availability to developmental regulation [27].

Carbohydrate metabolism also controls key developmental transitions such as flowering initiation and seed development. Trehalose-6-phosphate acts as a central metabolic signal that reflects cellular sucrose availability and regulates flowering time [28]. Experimental evidence shows that reduced trehalose-6-phosphate levels delay flowering in *Arabidopsis* by affecting the expression of the flowering regulator FLOWERING LOCUS T [29]. Amino acid metabolism further contributes to plant growth by supplying nitrogen and precursors for protein synthesis and secondary metabolism. Nitrogen assimilation pathways involving nitrate reductase and glutamine synthetase regulate biomass accumulation and root architecture in major crops such as maize and rice [30]. Lipid metabolism also plays a crucial role in plant development by supporting membrane biosynthesis, energy storage, and signaling processes associated with organ formation and cellular differentiation [31,32,33].

### 2.2. Hormonal Regulation of Metabolic Pathways

Plant hormones function as central regulators that connect metabolic status to developmental programs [34]. Auxin signaling controls cell elongation, tissue patterning, and organogenesis by transcriptionally regulating metabolic genes [35]. Auxin gradients influence carbohydrate metabolism by modulating sucrose transport and phloem unloading, ensuring that growing tissues receive sufficient metabolic resources. Studies using auxin-responsive mutants demonstrate that disruptions in auxin signaling frequently alter sugar metabolism and developmental growth patterns [36]. This coordination enables plants to direct metabolic flux toward actively developing organs such as young leaves, flowers, and roots.

Cytokinin, gibberellin, and brassinosteroid (BR) signaling further integrate metabolic pathways with developmental processes [37]. Cytokinins regulate cell division and shoot development while coordinating nitrogen metabolism and nutrient allocation. Experimental evidence indicates that cytokinin signaling enhances glutamine synthetase activity, promoting nitrogen assimilation and biomass accumulation [38]. Gibberellins play an essential role during seed germination by activating metabolic enzymes involved in starch degradation within the endosperm [39]. This process releases glucose and maltose, providing energy for seedling growth [40]. BR signaling also links nutrient availability with growth control. Under sugar-sufficient conditions, TOR signaling promotes S6K2-mediated phosphorylation of BIN2, which suppresses BIN2 activity and thereby enhances BR-responsive growth. Because BIN2 is a major negative regulator of BR signaling, its inhibition permits BR-dependent transcriptional programs that support cell expansion and anabolic growth [41,42,43]. The interaction between hormone signaling and metabolism therefore ensures that developmental processes occur only when sufficient metabolic resources are available.

### 2.3. Metabolic Checkpoints in Developmental Transitions

Plant development is tightly regulated by metabolic checkpoint systems that monitor cellular energy status and environmental conditions [44]. One of the most important metabolic sensors in plants is the SNF1-related kinase 1 (SnRK1) complex, which becomes activated under energy deficiency [45]. SnRK1 regulates transcriptional networks that suppress anabolic processes and promote catabolic pathways that generate energy [46]. Activation of SnRK1 therefore limits growth under nutrient deprivation while enhancing metabolic pathways associated with stress tolerance [46]. SnRK1 signaling is a major determinant of developmental plasticity under environmental stress [46].

Another key regulator of plant metabolic growth control is the target of rapamycin (TOR) kinase signaling pathway. TOR integrates nutrient availability, energy status, and hormonal signals to promote cell proliferation and protein synthesis [47]. Activation of TOR stimulates ribosome biogenesis, translation initiation, and metabolic pathways supporting rapid cellular growth [48]. Conversely, inhibition of TOR signaling reduces meristem activity and delays plant development. Environmental factors such as light intensity, temperature, and nutrient availability further influence these metabolic checkpoints [49]. Beyond its developmental functions, TOR is increasingly recognized as a central regulator linking hormonal stress signaling to the coordination of growth and defense.

## 3. Metabolic Regulation of Plant Defense Responses

Plants rapidly reprogram metabolism when exposed to pathogens or environmental stress [50]. Defense activation requires large metabolic investments because plants must synthesize antimicrobial compounds, signaling molecules, and structural barriers. Consequently, metabolic flux shifts from growth-related biosynthesis toward specialized metabolite production and immune signaling pathways [51]. Primary metabolism supplies precursors and energy required for defense metabolite synthesis. Carbohydrate metabolism, amino acid biosynthesis, and lipid pathways therefore become tightly linked with plant immune responses. These metabolic changes enable plants to produce defensive compounds such as phytoalexins, phenylpropanoids, and terpenoids that inhibit pathogen growth [7]. At the same time, metabolic reprogramming supports signaling pathways that coordinate systemic immune responses throughout plant tissues (Table 1). For clarity, defense-associated pathways can be broadly grouped into primary metabolic pathways that supply energy and biosynthetic precursors, secondary (specialized) metabolic pathways that generate antimicrobial compounds, and integrative defense-associated modules that coordinate redox regulation and systemic immunity.

### 3.1. Stress-Induced Metabolic Reprogramming

Plant immune activation triggers extensive metabolic adjustments that redistribute cellular resources toward defense responses [15,65]. Pathogen recognition typically occurs through pattern-recognition receptors that detect microbial molecules known as pathogen-associated molecular patterns. Activation of these receptors initiates signaling cascades that alter metabolic gene expression and redirect carbon flux toward defense pathways [66]. One early metabolic response involves enhanced glycolysis and the tricarboxylic acid cycle, which generate ATP and metabolic intermediates required for defense compound biosynthesis [67].

Pathogen infection is associated with increased flux through the shikimate pathway, which generates aromatic amino acids such as phenylalanine [68]. Phenylalanine acts as a precursor for numerous defense metabolites synthesised through the phenylpropanoid pathway. Increased shikimate pathway activity has been observed following infection with *Pseudomonas syringae*, highlighting the role of central metabolism in supporting immune responses [69]. Additionally, metabolic pathways involved in lipid oxidation are activated during pathogen attack, generating signaling molecules that regulate defense gene expression [70].

Metabolic reprogramming also involves the accumulation of reactive oxygen species and defense-related metabolites. The oxidative burst triggered during immune activation stimulates metabolic pathways that produce antimicrobial compounds and signaling molecules [71]. These metabolic adjustments enable plants to rapidly produce defense metabolites while maintaining essential cellular functions.

### 3.2. Secondary Metabolites in Plant Immunity

Secondary metabolites are crucial components of plant defense strategies. These compounds have antimicrobial, antioxidant, and signaling properties that help plants fend off pathogens and environmental stresses [72]. The phenylpropanoid pathway produces many defense metabolites, including lignin, flavonoids, and coumarins [73]. The shikimate pathway functions as the upstream metabolic route that supplies aromatic amino acid precursors for phenylpropanoid and phytoalexin biosynthesis during plant defense responses [74]. Lignin deposition and callose accumulation reinforce plant cell walls and help block pathogen entry, while flavonoids provide antimicrobial effects against fungi and bacteria [75]. Phytoalexins are another key group of defense metabolites generated during pathogen attacks. These substances quickly accumulate after infection and inhibit microbial growth [76]. In Arabidopsis, the indole phytoalexin camalexin plays a vital role in resisting fungal pathogens [77]. Camalexin is produced from tryptophan metabolism and is strongly induced during immune responses [77]. Similar phytoalexins have been identified in many crop species, including rice and soybean. Terpenoids and alkaloids also play important roles in plant immunity. Terpenoids participate in both direct and indirect defense by attracting beneficial organisms that reduce threats from herbivores or pathogens [72]. Alkaloids such as nicotine and morphine derivatives exhibit potent antimicrobial activity and help protect plants from herbivores [78]. These diverse classes of metabolites highlight the complexity of plant chemical defense systems.

### 3.3. Growth–Defense Trade-Off in Plant Metabolism

Activation of defense responses often imposes high metabolic costs on plant growth. Production of specialised metabolites requires substantial amounts of carbon, nitrogen, and energy that might otherwise support growth and reproduction [79]. Consequently, plants must carefully balance metabolic allocation between growth processes and defense mechanisms. This balance is commonly described as the growth–defense trade-off.

Hormonal signaling pathways play a critical role in regulating this metabolic balance. Salicylic acid (SA) primarily mediates defense responses against biotrophic pathogens, while jasmonic acid regulates responses to herbivores and necrotrophic pathogens [80]. Activation of these pathways frequently suppresses growth-related metabolic processes, thereby allowing plants to prioritise the production of defense compounds. For example, increased SA signaling can reduce photosynthetic activity and growth rate while enhancing pathogen resistance [81].

Recent studies suggest that metabolic regulators, such as the TOR and SnRK1 signaling pathways, also influence the growth–defense balance [82]. Under favorable non-stress conditions, TOR kinase phosphorylates PYL/RCAR ABA receptors, thereby suppressing ABA signaling and sustaining anabolic growth. In contrast, under biotic or abiotic stress conditions, ABA-activated SnRK2 kinases phosphorylate RAPTOR1B, a core component of the TOR complex, thereby inhibiting TOR and repressing growth. This reciprocal TOR–ABA regulatory module provides a mechanistic framework for understanding how plants dynamically shift metabolic resources from growth to stress adaptation and defense in response to environmental changes [83]. In addition, lipid metabolism is closely integrated with immune hormone homeostasis during plant defense. Beyond serving as a source of oxylipins and jasmonates, fatty acid metabolic components can influence the balance between salicylic acid- and jasmonic acid-associated responses. For example, Arabidopsis Acyl Carrier Protein 1 (ACP1) has been implicated in PAMP-triggered immunity, highlighting how fatty acid metabolism contributes to immune regulation and defense hormone coordination during stress [84].

## 4. Integration of Signaling Networks and Metabolic Reprogramming

**Figure 2:**
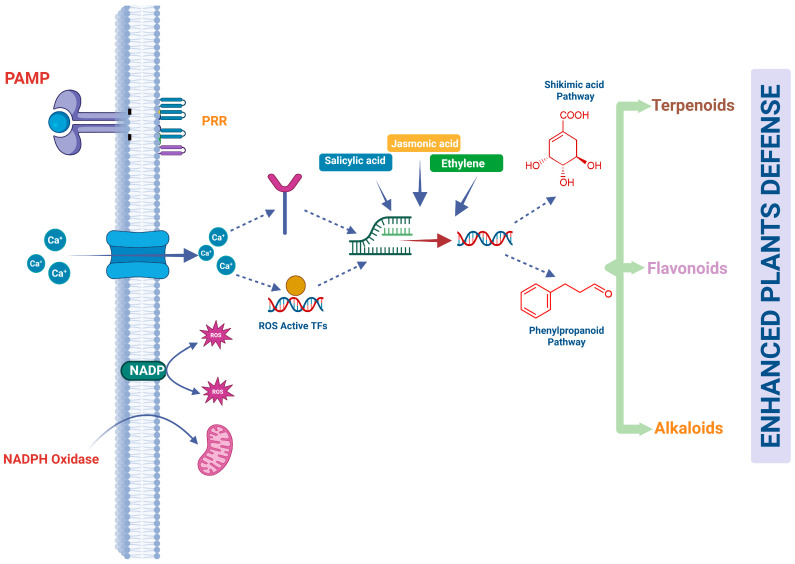
Conceptual Integration of signaling pathways and metabolic reprogramming during plant immune responses. Model illustrating how pathogen perception activates calcium, reactive oxygen species, and phytohormone signaling pathways, collectively remodeling central and specialized metabolism. These signaling networks redirect cellular resources from growth-related processes toward the production of defense metabolites, redox regulation, and local and systemic immune responses. Created in BioRender. Zaman, W. (2026) https://BioRender.com/04tpr26.

Plant defense responses rely on complex signaling networks that coordinate metabolic reprogramming throughout plant tissues. When plants perceive microbial molecules through pattern-recognition receptors, a cascade of intracellular signals rapidly activates transcriptional and metabolic responses. These signaling pathways regulate enzymes that control metabolic flux, enabling plants to synthesise antimicrobial compounds and structural defenses [72]. Central signaling components include calcium influx, production of reactive oxygen species, and phytohormone signaling networks [8]. These pathways function as regulatory hubs that integrate environmental perception with metabolic regulation. Through these networks, plants can rapidly redirect carbon and nitrogen metabolism toward the biosynthesis of defense compounds, while also reshaping flux through central pathways such as glycolysis, the tricarboxylic acid cycle, amino acid metabolism, and the shikimate pathway to support stress adaptation and immune output [85]. Signaling-driven metabolic regulation also coordinates systemic responses, allowing distal tissues to prepare for potential pathogen invasion [86]. The integration of signaling and metabolism therefore forms the molecular basis of plant immunometabolism (Figure 2). More specifically, immune signaling and metabolic reprogramming are coupled at multiple regulatory levels. Early calcium influx and ROS accumulation do not merely activate defense genes; they also influence enzyme activity, redox homeostasis, and the availability of metabolic intermediates required for defense. These early events promote the redistribution of resources from growth-associated metabolism toward the synthesis of phenylpropanoids, phytoalexins, terpenoids, and other defense-related compounds. Hormonal networks such as salicylic acid, jasmonic acid, ethylene, abscisic acid, and brassinosteroids further refine this response by coordinating transcriptional and post-translational regulation of key biosynthetic pathways. In this way, immune signaling acts not simply as an upstream trigger, but as an active organizer of metabolic flux, linking pathogen perception with local defense activation, systemic signaling, and the broader growth–defense trade-off.

### 4.1. Calcium and Reactive Oxygen Species Signaling

Calcium signaling is among the earliest events following pathogen recognition in plants. Pattern recognition receptors located on the plasma membrane detect pathogen-associated molecular patterns such as bacterial flagellin or fungal chitin [87]. Recognition of these molecules triggers rapid opening of calcium channels in the plasma membrane and endomembrane systems [88]. This process causes transient increases in cytosolic calcium concentration within seconds of pathogen perception [89]. Calcium ions act as secondary messengers, activating calcium-dependent protein kinases and calmodulin-regulated transcription factors [90]. These proteins regulate the expression of genes involved in defense metabolism, including enzymes of the phenylpropanoid and phytoalexin biosynthetic pathways. Calcium-dependent protein kinases are involved in regulating the transcription of phenylalanine ammonia-lyase and other enzymes linked to lignin biosynthesis [91]. Increased lignin deposition, together with callose deposition, strengthens cell walls and restricts pathogen penetration.

Reactive oxygen species (ROS) signaling closely interacts with calcium signaling during plant immune activation [8,92]. Shortly after pathogen perception, plants produce an oxidative burst characterised by the rapid accumulation of hydrogen peroxide and superoxide radicals [93]. These ROS molecules are generated primarily by plasma membrane NADPH oxidases known as respiratory burst oxidase homologs (RBOHs) [94]. Hydrogen peroxide diffuses across cell walls and membranes, acting as both an antimicrobial compound and a signaling molecule [95]. ROS signaling stimulates transcription factors that regulate metabolic pathways, producing defense metabolites. For example, ROS accumulation enhances expression of genes involved in flavonoid and phenolic compound biosynthesis [96]. These metabolites exhibit strong antimicrobial activity and contribute to plant resistance against fungal and bacterial pathogens.

Calcium and ROS pathways often operate through positive feedback mechanisms that amplify defense signaling [97]. Calcium influx can activate NADPH oxidases, thereby increasing ROS production. In turn, ROS molecules influence calcium channel activity, sustaining intracellular calcium oscillations [98]. This feedback loop strengthens immune signaling and ensures robust metabolic responses during pathogen attack. Such coordinated signaling events enable plants to rapidly mobilise metabolic resources and synthesise defense compounds required for effective immune protection.

### 4.2. Hormonal Crosstalk in Plant Immunity

Plant hormones play essential roles in integrating immune signaling with metabolic responses. Among these hormones, SA is particularly important for defense against biotrophic pathogens that require living host tissues [99]. SA accumulates in infected tissues and activates transcription factors that regulate the expression of defense genes. One key regulatory protein is nonexpressor of pathogenesis-related genes 1 (NPR1)**,** which controls transcription of numerous immune-related genes [100]. Activation of SA signaling also stimulates metabolic pathways that produce phenylpropanoid compounds such as lignin and flavonoids. These metabolites strengthen plant tissues and inhibit pathogen proliferation.

Jasmonic acid represents another major hormonal regulator of plant defense metabolism. Jasmonate signaling is particularly important for responses against necrotrophic pathogens and herbivorous insects [101]. Biosynthesis of jasmonic acid begins with the oxidation of α-linolenic acid derived from chloroplast membrane lipids. Enzymes such as lipoxygenases and allene oxide synthase catalyse the formation of jasmonate precursors [102]. Activation of jasmonate signaling stimulates metabolic pathways producing terpenoids, alkaloids, and proteinase inhibitors that deter herbivores and pathogens. For example, jasmonate signaling strongly induces nicotine biosynthesis in tobacco plants, providing chemical protection against insect herbivores [103].

Ethylene signaling interacts with both SA and jasmonic acid pathways to coordinate plant immune responses. Crosstalk among these hormonal pathways determines the outcome of plant–pathogen interactions [104]. Ethylene can enhance jasmonate-mediated defense responses while suppressing certain SA pathways [105]. Brassinosteroids also participate in this immune crosstalk. During pathogen infection, BR signaling enhances SA-mediated defense responses by suppressing BIN2-catalyzed phosphorylation of clade I TGA transcription factors in Arabidopsis. Because BIN2 phosphorylation weakens TGA function, BR-dependent BIN2 inactivation promotes TGA–NPR1-associated SA signaling and stronger defense gene expression. These interactions allow plants to tailor metabolic responses according to the type of pathogen encountered. Through these hormone-regulated networks, plants dynamically adjust metabolic pathways that control the synthesis of defense metabolites [13,106,107].

### 4.3. Systemic Signaling and Metabolic Coordination

Plant immune responses often extend beyond the initial infection site through systemic signaling mechanisms. One of the best-characterised systemic defense strategies is systemic acquired resistance (SAR) [108]. SAR enables plants to establish long-lasting resistance against a broad spectrum of pathogens following localised infection. During SAR activation, mobile signaling molecules travel through vascular tissues, activating defense gene expression in distant organs [109]. This process prepares uninfected tissues for potential pathogen attack by stimulating metabolic pathways that produce antimicrobial compounds.

Several metabolites have been identified as systemic signaling molecules involved in SAR. One important mobile signal in SAR is methyl salicylate, a volatile derivative of salicylic acid that can move through the phloem and be converted back into salicylic acid in distant organs [110]. Another systemic signal is azelaic acid, a lipid-derived molecule that primes immune responses in distal tissues. Azelaic acid enhances the expression of genes involved in SAbiosynthesis and defense metabolism [111]. These signaling molecules coordinate systemic metabolic responses that enhance the plant’s overall defensive capacity.

In addition to chemical signals, plants also use electrical and calcium waves to transmit defense signals across tissues [112]. Mechanical damage or pathogen infection can trigger electrical signals that propagate through vascular tissues at rapid speeds. These signals stimulate calcium influx and activate metabolic enzymes associated with stress responses [113]. Through these combined signaling mechanisms, plants synchronise metabolic reprogramming throughout the organism, ensuring coordinated defense against environmental threats. Taken together, these observations show that immune signaling and metabolic reprogramming are tightly interdependent processes. Calcium and ROS function as rapid early signals, phytohormones refine pathway specificity according to stress type, and systemic mobile signals extend these responses beyond the infection site. Through this multilayered regulatory architecture, plants coordinate central metabolism, redox balance, and defense metabolite biosynthesis across tissues, enabling an integrated transition from basal growth programs to local and systemic immunity.

## 5. Multi-Omics Approaches to Study Plant Immunometabolism

**Figure 3:**
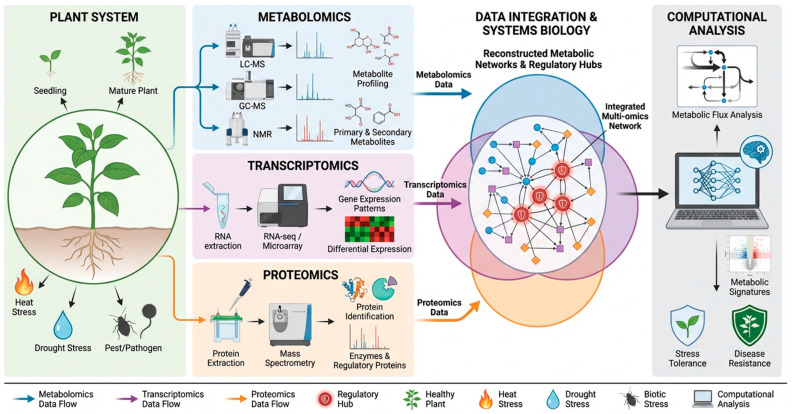
Multi-omics framework for investigating plant immunometabolism. Conceptual framework showing how representative metabolomics, transcriptomics, proteomics, and computational analyses can be integrated to resolve candidate the regulatory networks underlying plant immunometabolism. Integration of these datasets enables the identification of metabolic pathways, signaling hubs, and candidate regulators associated with development, stress adaptation, and defense under both biotic and abiotic stress conditions. The workflow is illustrative and does not represent an exhaustive or strictly linear analytical pipeline. Created in BioRender. Zaman, W. (2026) https://BioRender.com/04tpr26.

Advances in high-throughput technologies have transformed the study of plant metabolic regulation during development and immune responses. Traditional biochemical approaches provided valuable information about individual metabolites, yet they could not capture the complexity of plant metabolic networks operating under stress conditions. Modern metabolomics and multi-omics technologies now allow comprehensive profiling of thousands of metabolites and regulatory molecules simultaneously [114]. These approaches enable researchers to reconstruct metabolic pathways and identify regulatory hubs that control plant defense responses. Integration of metabolomics with transcriptomics, proteomics, and epigenomics has revealed complex interactions between metabolic pathways and gene regulatory networks [115]. Systems-level studies demonstrate that metabolic reprogramming during plant stress responses involves coordinated regulation of biosynthetic enzymes, transcription factors, and signaling molecules [116]. These advances have laid the foundation for the emerging field of plant immunometabolism, which investigates how metabolic networks interact with immune signaling pathways to determine plant resistance or susceptibility to environmental stress (Figure 3).

### 5.1. Metabolomics Technologies in Plant Biology

Metabolomics represents a powerful analytical approach for identifying and quantifying metabolites produced by plants under different physiological conditions. Modern metabolomics platforms rely primarily on mass spectrometry and NMR technologies. LC-MS is widely used because it enables the detection of a broad range of metabolites, including phenolics, alkaloids, flavonoids, and phytohormones [117]. GC-MS is particularly effective for analysing volatile metabolites and primary metabolic intermediates such as sugars, amino acids, and organic acids [118]. Nuclear magnetic resonance spectroscopy provides complementary structural information and allows absolute quantification of metabolites without extensive sample preparation.

Large-scale metabolomic analyses have revealed extensive metabolic changes during plant defense responses. LC–MS-based metabolomic analyses have identified marked changes in metabolite abundance during immune activation [119]. These metabolites included phenylpropanoid derivatives, glucosinolates, and phytoalexins that are involved in antimicrobial defense [120]. Similar metabolomic studies in rice exposed to fungal pathogens demonstrated strong induction of diterpenoid phytoalexins such as momilactones and phytocassanes [121]. These compounds accumulate rapidly in infected tissues and inhibit fungal growth. Such studies highlight the ability of metabolomics technologies to uncover previously unknown metabolic pathways involved in plant immunity.

Metabolomics also plays a critical role in understanding metabolic regulation during abiotic stress. Environmental stresses such as drought, salinity, and temperature extremes alter plant metabolic profiles by modifying carbohydrate metabolism, amino acid biosynthesis, and antioxidant pathways [122]. For example, drought stress often increases the accumulation of osmoprotective metabolites, including proline, glycine betaine, and soluble sugars [123]. These compounds protect cellular structures and maintain osmotic balance under water-limited conditions. Comprehensive metabolomic profiling therefore provides essential insights into how plants adapt metabolically to environmental challenges.

### 5.2. Integration of Multi-Omics Datasets

While metabolomics provides direct information about biochemical compounds, integrating it with other omics technologies enables a deeper understanding of the regulatory mechanisms that control metabolic networks. Transcriptomics reveals gene expression patterns associated with metabolic pathway activation [124]. Proteomics identifies enzymes and regulatory proteins responsible for metabolite biosynthesis [125]. Combining these datasets enables researchers to reconstruct complete metabolic networks and identify regulatory nodes that control plant immunity.

Multi-omics integration has been particularly useful for understanding complex defense pathways such as the phenylpropanoid biosynthetic network. Transcriptomic studies have identified numerous transcription factors regulating phenylpropanoid metabolism during pathogen infection [126]. These transcription factors control the expression of enzymes such as PAL, cinnamate-4-hydroxylase, and chalcone synthase [127]. Proteomic analyses confirm the presence of these enzymes in infected tissues and reveal post-translational modifications that influence their catalytic activity [128]. When combined with metabolomic measurements of flavonoids and lignin precursors, these datasets provide a comprehensive view of metabolic pathway regulation during immune responses.

Network-based analysis of multi-omics datasets also enables the identification of regulatory hubs that coordinate plant defense metabolism [129]. Computational models often reveal highly connected genes or enzymes that control metabolic flux through multiple pathways. For instance, systems biology studies have identified transcription factors such as WRKY, MYB, and bHLH families as central regulators of plant defense metabolism [130,131]. These transcription factors control the expression of numerous metabolic enzymes involved in phenylpropanoid, terpenoid, and alkaloid biosynthesis. Multi-omics integration therefore enables the discovery of key regulatory components governing plant immunometabolism.

### 5.3. Computational Modelling of Plant Metabolic Networks

Computational approaches have become essential for interpreting complex multi-omics datasets generated from plant systems. Metabolic network modelling enables researchers to predict metabolic flux distributions under different environmental conditions [132]. Genome-scale metabolic models integrate biochemical reactions, gene expression data, and metabolite measurements to simulate metabolic behaviour in plant cells [133]. These models help identify metabolic bottlenecks and regulatory nodes that influence plant stress tolerance.

One widely used modelling approach is flux balance analysis, which estimates metabolic flux through biochemical pathways based on stoichiometric constraints and metabolic demand [134]. In *Arabidopsis*, genome-scale metabolic models have been used to simulate metabolic responses to pathogen infection and nutrient stress [135]. These models revealed that defense activation requires increased flux through the shikimate and phenylpropanoid pathways [136]. Such predictions are consistent with experimental metabolomic data showing enhanced production of phenylpropanoid defense metabolites during pathogen attack.

Machine learning approaches are also increasingly applied to plant metabolomics datasets. Algorithms capable of analysing high-dimensional metabolomic data can identify metabolic signatures associated with stress tolerance or disease resistance [137]. For example, machine learning models trained on metabolomic profiles of rice varieties have successfully predicted resistance to fungal pathogens [138]. These computational tools provide powerful frameworks for analysing plant metabolic complexity and identifying key metabolic traits that could be targeted in crop improvement programs.

## 6. Agricultural and Biotechnological Applications

Understanding plant immunometabolism has important implications for improving crop resilience and agricultural sustainability [139]. Plants continuously experience biotic and abiotic stresses that significantly reduce global crop productivity. Traditional breeding strategies have improved resistance in many crop species, yet these approaches often rely on single resistance genes that pathogens can quickly overcome [140]. Recent research indicates that manipulating metabolic pathways provides a complementary strategy for enhancing plant defense capacity. By modifying key metabolic nodes, it is possible to increase production of antimicrobial compounds, strengthen structural barriers, and enhance stress tolerance without compromising growth [141]. Advances in genomics, genome editing, and metabolic engineering now enable precise manipulation of metabolic networks that control plant defense responses [142]. These technologies are increasingly being applied to develop crop varieties capable of maintaining productivity under pathogen pressure and environmental stress conditions (Table 2).

### 6.1. Metabolic Engineering for Crop Resistance

Metabolic engineering has emerged as a powerful strategy for enhancing plant resistance to pathogens by modifying biosynthetic pathways that produce defense metabolites [157]. Many defense-related compounds originate from the phenylpropanoid pathway, which generates lignin, flavonoids, and antimicrobial phenolic compounds [73]. Engineering key enzymes within this pathway can significantly enhance plant resistance. For example, overexpression of PAL in several plant species has been shown to increase lignin deposition and improve resistance against fungal pathogens [158]. Similarly, increased expression of chalcone synthase enhances flavonoid biosynthesis, strengthening plant defense mechanisms.

Terpenoid metabolic pathways also represent promising targets for engineering plant resistance. Terpenoids include a diverse class of secondary metabolites that function as antimicrobial compounds and signaling molecules [159]. In rice, genetic modification of diterpenoid biosynthesis pathways has increased production of phytoalexins such as momilactones and phytocassanes [121]. These compounds exhibit strong antifungal activity against pathogens such as *Magnaporthe oryzae*, the causative agent of rice blast disease. Engineering terpenoid metabolism therefore offers a promising strategy for improving disease resistance in cereal crops [160].

Metabolic engineering approaches have also been applied to enhance alkaloid production in medicinal and crop plants [161]. Alkaloids often possess potent antimicrobial properties and contribute to plant defense against herbivores. For instance, manipulation of nicotine biosynthesis in tobacco plants has demonstrated that elevated alkaloid levels can significantly increase resistance to insect herbivores [162]. These examples illustrate how metabolic pathway engineering can strengthen plant defense systems by enhancing the biosynthesis of protective compounds.

### 6.2. Genome Editing Approaches

Genome editing technologies have revolutionised plant biotechnology by enabling precise modification of genes that control metabolic pathways [163]. Among these technologies, the CRISPR–Cas system has become widely used because of its efficiency, versatility, and relative simplicity [164]. CRISPR-based editing enables targeted modification of genes encoding metabolic enzymes, transcription factors, and signaling proteins involved in plant defense responses [165]. Such modifications can alter metabolic flux through key pathways, thereby enhancing plant resistance to pathogens and environmental stress.

Several studies have successfully used CRISPR-mediated genome editing to modify plant metabolic pathways associated with immunity. In plants, CRISPR editing of genes regulating jasmonic acid signaling has improved resistance against insect herbivores by increasing production of defense-related metabolites [166]. Similarly, editing of transcription factors controlling phenylpropanoid biosynthesis has enhanced disease resistance in several crop species [167]. Because these transcription factors regulate multiple metabolic enzymes simultaneously, modifying them can produce broad metabolic changes that strengthen plant immunity.

Genome editing also offers opportunities to reduce susceptibility factors that pathogens exploit during infection. Certain plant metabolic pathways produce molecules required for pathogen growth or colonisation [168]. Disrupting these pathways through targeted gene editing can limit pathogen access to essential nutrients. For example, modification of sugar transporters that supply carbohydrates to pathogens has improved disease resistance in sweet sugar in rice and wheat [169,170]. Such strategies demonstrate how genome editing can reshape plant metabolic networks to favour defense responses.

### 6.3. Metabolic Markers for Crop Breeding

Metabolomic profiling has become an increasingly valuable tool for identifying metabolic traits associated with stress tolerance and disease resistance [171]. Traditional breeding programs rely primarily on phenotypic selection, which can be time-consuming and influenced by environmental variability [172]. Metabolomic approaches allow breeders to identify biochemical markers that correlate with desirable agronomic traits [173]. These metabolic markers can be used to screen large breeding populations and accelerate the development of stress-resilient crop varieties.

Several metabolite classes have been identified as indicators of plant stress tolerance. Elevated levels of phenylpropanoid compounds often correlate with enhanced pathogen resistance because these metabolites strengthen cell walls and possess antimicrobial activity [174]. Similarly, increased accumulation of osmoprotective metabolites such as proline and glycine betaine is associated with improved tolerance to drought and salinity stress [175]. By monitoring these metabolites, breeders can identify plant genotypes that exhibit superior stress adaptation.

Metabolomics-guided breeding programs are already being applied in major crops, including rice, maize, and wheat [176]. These approaches combine metabolite profiling with genomic selection to identify genes controlling beneficial metabolic traits. Integration of metabolomic data with genomic and transcriptomic information enables breeders to pinpoint candidate genes that regulate stress-responsive metabolic pathways [176]. Such integrative strategies represent a promising avenue for developing crops that maintain productivity under increasingly challenging environmental conditions.

## 7. Conceptual Framework of Plant Immunometabolism

**Figure 4:**
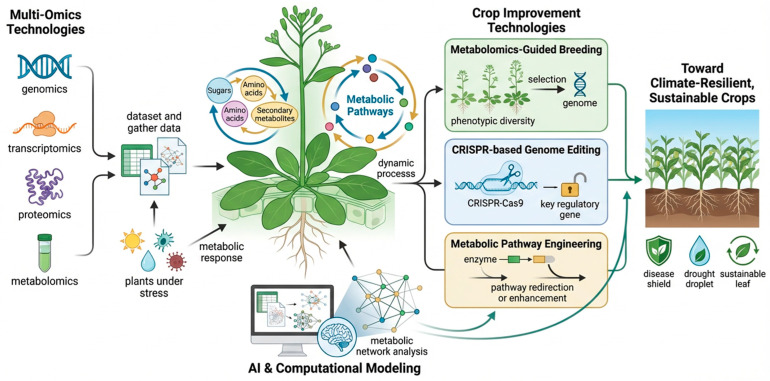
Future research roadmap for plant immunometabolism and crop improvement. Summary of emerging directions for advancing plant immunometabolism research and its agricultural application. The figure highlights priorities including integrative multi-omics, predictive modeling, artificial intelligence-assisted analysis, metabolic engineering, genome editing, and metabolomics-guided breeding for the development of climate-resilient crops.Created in BioRender. Zaman, W. (2026) https://BioRender.com/04tpr26.

Plant immunometabolism offers a comprehensive framework illustrating how metabolic networks coordinate plant growth, development, and immune responses [177]. Instead of operating as isolated pathways, plant metabolism functions within interconnected regulatory networks that adaptively respond to environmental cues. Under optimal conditions, these pathways mainly support growth by providing energy, carbon skeletons, and biosynthetic intermediates necessary for cell proliferation and tissue development [18]. When plants face pathogens or stress, these networks reprogram to divert resources toward defense mechanisms, creating a balance between growth and defense that underpins plant adaptive strategies. Within this system, immune signaling pathways act as switches that regulate metabolic flow. Detection of pathogen-associated molecules triggers signaling cascades involving calcium, reactive oxygen species, and phytohormones [178]. These signals modify gene expression and enzyme activity, reshaping metabolic networks to produce defense compounds and stress-related metabolites. Thus, metabolic pathways serve as both sources of defense molecules and regulators that link environmental signals to physiological responses. Recent advances in multi-omics technologies have revealed the highly interconnected nature of these networks. Combining metabolomics, transcriptomics, and proteomics shows that plant metabolic responses are tightly coordinated across multiple biological layers [11]. These insights position metabolic networks as central hubs that connect environmental sensing, immune signaling, and adaptation. Understanding this integrated regulatory system opens new avenues for enhancing crop resilience through targeted metabolic engineering, genome editing, and metabolomics-guided breeding. As shown in Figure 4, the concept of plant immunometabolism serves as a unifying framework that connects plant metabolism, immune signaling, and agricultural innovation.

## 8. Future Perspectives and Emerging Research Directions

Despite major advances in plant metabolic research, many questions remain regarding how metabolic networks coordinate plant development and immune responses. Current studies have identified numerous metabolites and signaling molecules involved in plant defense [64], yet the regulatory relationships among these pathways remain only partially understood. Plant metabolism operates through highly interconnected biochemical networks that respond dynamically to environmental signals [179]. Understanding these complex interactions requires integrative approaches that combine metabolomics, genomics, and computational modelling. The emerging field of plant immunometabolism provides a conceptual framework for studying how metabolic pathways influence plant growth–defense trade-offs [180]. Continued research in this area will improve our understanding of how plants allocate metabolic resources between development and defense under changing environmental conditions. Such knowledge is essential for developing crops capable of maintaining productivity under increasing biotic and abiotic stress pressures (Figure 4).

### 8.1. Integrating Multi-Omics for Predictive Plant Biology

Future research on plant immunometabolism will increasingly rely on integrated multi-omics approaches that capture the complexity of metabolic regulation. While metabolomics provides detailed information about biochemical compounds present in plant tissues, combining metabolomic data with transcriptomic and proteomic analyses enables the reconstruction of complete regulatory networks that control metabolic flux [181]. These integrated datasets allow researchers to identify key transcription factors, enzymes, and signaling molecules that regulate plant metabolic responses to environmental stress.

Large-scale multi-omics studies have already begun revealing how plant metabolic pathways respond to pathogen infection and environmental stress [182]. For example, integrated transcriptomic and metabolomic analyses in rice exposed to fungal pathogens have identified coordinated activation of genes controlling phenylpropanoid metabolism and diterpenoid phytoalexin biosynthesis [183]. Such studies demonstrate that metabolic responses to stress involve tightly coordinated regulation across multiple biological layers. Expanding these approaches across diverse crop species will help identify conserved metabolic mechanisms that control plant defense. Future efforts will also focus on developing predictive models that link metabolic network activity with plant physiological outcomes. Integrating multi-omics data with physiological measurements such as growth rate, photosynthetic efficiency, and stress tolerance will enable researchers to identify metabolic signatures associated with desirable agronomic traits. These predictive frameworks could guide crop-breeding and metabolic-engineering strategies aimed at improving plant resilience.

### 8.2. Artificial Intelligence in Plant Metabolic Research

Artificial intelligence and machine learning approaches are rapidly transforming the analysis of large biological datasets generated through multi-omics technologies [184]. Plant metabolomic datasets often contain thousands of variables representing diverse metabolites that interact through complex biochemical networks. Traditional statistical approaches struggle to capture nonlinear relationships among these variables. Machine learning algorithms, however, can analyse high-dimensional datasets and identify patterns associated with stress responses or disease resistance [185]. Several recent studies have applied machine learning methods to classify plant metabolic responses under different environmental conditions. For example, machine learning models trained on metabolomic datasets from rice varieties have successfully predicted resistance to fungal pathogens by identifying metabolic markers associated with defense responses [186]. Similar approaches have been used to analyse metabolomic responses of maize to drought stress, revealing key metabolic pathways that contribute to stress tolerance. These studies illustrate the potential of artificial intelligence to uncover previously unknown metabolic relationships within plant systems [187]. Artificial intelligence tools can also assist in reconstructing metabolic networks by integrating information from genomic, transcriptomic, and metabolomic datasets. Network inference algorithms can identify regulatory interactions between transcription factors and metabolic enzymes, enabling prediction of metabolic pathway behaviour under different environmental scenarios [188]. As computational tools continue to improve, artificial intelligence will become an essential component of plant metabolic research.

### 8.3. Toward Climate-Resilient Crops

Climate change is expected to intensify environmental stresses affecting global agricultural productivity. Rising temperatures, irregular rainfall patterns, and increased pathogen pressure threaten food security in many regions of the world [189]. Understanding plant immunometabolism presents an important opportunity to develop crops that can withstand these environmental challenges. By identifying metabolic pathways that enhance stress tolerance, researchers can design strategies to improve plant resilience without compromising productivity.

Crop improvement programs are increasingly incorporating metabolic traits associated with stress tolerance into breeding strategies. For example, accumulation of osmoprotective metabolites such as proline and soluble sugars improves plant tolerance to drought and salinity stress [190]. Similarly, enhanced production of phenylpropanoid compounds strengthens cell walls and increases resistance to pathogens [174]. Identifying genetic regulators controlling these metabolic traits allows breeders to develop crop varieties that maintain productivity under adverse environmental conditions [191]. Future crop improvement efforts will likely combine metabolic engineering, genome editing, and metabolomics-guided breeding strategies. Integrating these approaches will enable precise modification of plant metabolic pathways to optimise the balance between growth and defense under variable environmental conditions. Such innovations will be critical for developing sustainable agricultural systems capable of meeting the food demands of a growing global population.

## 9. Conclusions

Plant metabolism is essential for coordinating growth, development, and defense in changing environments. Throughout a plant’s life cycle, metabolic pathways supply energy, biosynthetic precursors, and signaling molecules needed for development. Plants must also quickly modify these networks in response to pathogen attacks or environmental stress to activate immune responses. This involves extensive metabolic reprogramming that redirects cellular resources toward the production of defense compounds and toward adaptation to stress. Recent progress in metabolomics and multi-omics technologies has deepened our understanding of how metabolism influences plant immunity. Studies that integrate various omics reveal that stress responses are governed by complex networks involving transcription factors, signaling molecules, and biosynthetic enzymes. Pathways such as calcium signaling, reactive oxygen species production, and phytohormone networks coordinate metabolic changes that enhance plant resistance to pathogens and stress. The concept of plant immunometabolism offers a useful framework for understanding how metabolism and immune signaling interact dynamically. By linking metabolic networks, signaling pathways, and growth processes, this framework explains how plants balance growth and defense amid environmental fluctuations. Advances in genome editing, metabolic engineering, and metabolomics-guided breeding highlight the potential to manipulate these pathways to enhance crop resilience. Future research combining systems biology, AI, and advanced metabolomics will yield deeper insights into metabolic regulation, supporting the development of crops capable of sustaining productivity under stress. Overall, understanding plant immunometabolism is a key step toward sustainable agriculture and global food security.

## Figures and Tables

**Table 1:** Major metabolic pathways involved in plant defense responses.

Category	Pathway	Core Immune Role	Major Outputs/Markers	Why Is This Pathway Currently Important	References
Primary metabolism	Carbohydrate metabolism	Reallocates carbon and energy from growth to defense	Soluble sugars, glycolytic/TCA intermediates, carbon skeletons for defense metabolites	Central to the growth–defense trade-off and metabolic fueling of immunity	[52,53]
Primary metabolism	Amino acid metabolism	Generates defense signals and precursors for immune metabolites	Pipecolic acid (Pip), N-hydroxypipecolic acid (NHP), glutamate, proline, GABA	Highly relevant because amino acid-derived metabolites now sit at the centre of local and systemic immunity	[54,55]
Primary metabolism	Lipid metabolism	Produces membrane-derived immune signals	Phosphatidic acid, oxylipins, jasmonates, lipid remodelling products	A major signaling hub linking membranes, defense hormones, and immune activation; also increasingly recognized for its role in maintaining salicylic acid- and jasmonic acid-related immune homeostasis during plant defense	[56]
Primary metabolism	Shikimate pathway	Supplies chorismate and aromatic amino acid precursors for defense metabolism	Chorismate, phenylalanine, tyrosine, tryptophan; salicylic acid-associated precursors	Trendy because it connects primary metabolism directly to SA biosynthesis, phenylpropanoids, and pathogen effector targeting	[57,58]
Secondary (specialized) metabolism	Phenylpropanoid pathway	Produces structural and antimicrobial defenses, including reinforcement of the cell wall	Lignin, flavonoids, coumarins, phenolics, phytoalexins, callose deposition	Remains a flagship pathway in immunity because it links metabolic reprogramming with both cell wall reinforcement and chemical defense.	[59,60]
Secondary (specialized) metabolism	Terpenoid metabolism	Produces antimicrobial and volatile defense compounds	Terpenoid phytoalexins, diterpenes, sesquiterpenes, defense volatiles	Important for direct toxicity to pathogens and emerging multi-omics studies of induced defense chemistry	[61]
Integrative defense-associated metabolic/signaling modules	Reactive oxygen species (ROS) metabolism	Functions in oxidative burst, signaling, antimicrobial defense, and redox homeostasis	H_2_O_2_, O_2_^•−^, hydroxyl radicals, antioxidant enzymes (SOD, CAT, POD, APX)	One of the hottest areas in plant immunity is because ROS are now viewed as both signals and metabolic regulators, not just toxic by-products	[9,62]
Integrative defense-associated metabolic/signaling modules	Systemic defense metabolism	Coordinates long-distance immune signaling and priming	Methyl salicylate, pipecolic acid (Pip), N-hydroxypipecolic acid (NHP)	Essential in current immunity papers because systemic signaling and immune memory are major themes	[63,64]

**Table 2:** Biotechnological strategies targeting plant metabolic pathways for crop improvement.

Strategy	Target Pathway/System	Agricultural Benefit	Example Application	References
Phenylpropanoid pathway engineering	Phenylpropanoid metabolism	Strengthens structural barriers and increases antimicrobial metabolites	Enhanced lignin, callose deposition, flavonoid, and phenolic accumulation for pathogen resistance	[143]
Phytoalexin engineering	Specialised metabolite pathways	Increases inducible antimicrobial compound production	Enhanced camalexin and diterpenoid phytoalexin biosynthesis	[144,145]
Lipid signaling manipulation	Jasmonate/oxylipin pathways	Improves resistance to insects and necrotrophic pathogens	Increased jasmonate-dependent defense responses and improved immune regulation through lipid-mediated control of defense hormone homeostasis	[146]
Terpenoid pathway engineering	Terpenoid metabolism	Enhances antimicrobial metabolites and defense volatiles	Increased terpenoid phytoalexins and insect-repellent volatiles	[147,148]
Genome editing of metabolic regulators	Multiple defense-related pathways	Enables precise resistance enhancement with reduced growth penalty	CRISPR/Cas editing of pathway genes and regulatory nodes	[149,150,151]
Metabolic pathway stacking	Multiple defense and stress pathways	Confers broader and more durable resistance	Combined engineering of defense metabolites and signaling pathways	[152]
Transcription factor/regulatory network engineering	MYB, WRKY, NAC and related regulators	Activates multigene defense programs in a coordinated manner	Rewiring specialised metabolism through pathway-level regulators	[152,153]
ROS/antioxidant pathway engineering	Redox and ROS-scavenging metabolism	Reduces oxidative damage and improves abiotic stress resilience	Enhancement of antioxidant enzymes and redox-buffering metabolites	[154,155]
Synthetic biology and multi-omics-guided engineering	Whole metabolic networks	Accelerates pathway discovery and trait optimisation	Integrating genomics, metabolomics, DNA synthesis, and pathway design	[156]

## Data Availability

No new data were created or analyzed in this study.
